# The EU Industrial Strategy: Towards a Post-Growth Agenda?

**DOI:** 10.1007/s10272-021-0968-7

**Published:** 2021-06-04

**Authors:** Andrea Renda

**Affiliations:** Centre for European Policy Studies (CEPS), 1 Place du Congrès, 1000 Brussels, Belgium

## Abstract

Along with the Green Deal, the von der Leyen Commission immediately started to look at how to adopt an industrial strategy that would promote EU competitiveness and support the Commission’s self-assigned “geopolitical” role by boosting strategic autonomy.

The nature and intensity of EU industrial policy has drastically changed over the past decades. As a matter of fact, rather than engaging in industrial policy on its own, in its first few months, the European Community tried to prevent its member states from using industrial policy measures that could jeopardise the Single Market. Rules on state aid, for example, saw their first application in the 1960s in an attempt to stop member states from altering the competitive playing field through export aid for intra-community trade. Concerns related to Europe's inability to keep the pace of the US led to the first attempts to coordinate industrial policy. Many of these attempts, however, failed due to tensions between member states, and an overall hesitancy when it came to abandoning national prerogatives in favour of a more coordinated policy at the EU level (Pavitt, 1971).

After this first wave, the EU entered an "industrial policy winter": emphasis on completing the Single Market and the gradual implementation of rather *laissez-faire* economic approaches inspired by the US gradually transformed the words "industrial policy" into an oxymoron, increasingly taboo in EU-level public discourse. The Lisbon strategy set targets for R&D investment and employment, but fell short of laying the foundations of an effective industrial policy: by then, indeed, the term industrial policy had become largely *démodé*. The Lisbon strategy was critically affected by a lack of coordination and commitment by member states, leading to a significant dilution of the ambition with the "Kok report" in 2004. The following "Europe 2020" strategy, launched by the Barroso Commission in 2009, in the midst of the dramatic financial and economic crisis, refocused the EU agenda towards "smart, sustainable and inclusive growth". The strategy was so rapidly eroded by the unfolding post-crisis events that already in 2014, the incoming Juncker Commission decided not to review it, de facto replacing it with a more pragmatic focus on "ten priorities", which left very little space for a coordinated and ambitious industrial policy and was rather oriented towards doing less, more efficiently. Despite the attempt to trigger an "industrial renaissance" in Europe since 2014, results have remained rather meagre.

**Andrea Renda**, Centre for European Policy Studies, Brussels, Belgium; and European University Institute, Florence, Italy.

## The von der Leyen Commission: Departing from a growth-oriented narrative?

When the von der Leyen Commission took office in November 2019, the political attention shifted towards a more assertive European Commission, focused on so-called "competitive sustainability". Emphasis on the need to reorient policy efforts towards the Sustainable Development Goals (SDGs) resulted in the implementation of these goals in the European Semester as well as in external action, i.e. the launch of the European Green Deal, accompanied by the so-called "just transition", which increased the salience of social impacts alongside climate targets in the EU, although with an extremely narrow focus. The objective of achieving climate neutrality by 2050, later coupled with a resounding commitment to cut 55% of emissions by 2030, became the defining trait of the EU as an internal and global change actor. This move also echoed an existing trend at the global level, i.e. the gradual transition from growth-oriented policies towards goal-based policy, centred around the SDGs. This is a fundamental shift, evoked by several governments, activists and academics around the world, but only sparsely implemented in practice. The von der Leyen Commission's bold move to put sustainability upfront raised huge expectations among those who have advocated such transitions for decades.

At the same time, the resounding announcements on the Green Deal and the Just Transition Mechanism also overshadowed the lack of bold commitments on certain aspects of the SDGs, on which EU institutions have very weak competences. These include, notably, education, health, good governance and the rule of law – all fronts on which more will have to be done in the future.

## The 2020 Communication on the Industrial Strategy

Along with the Green Deal, the von der Leyen Commission immediately started to look at how to adopt an industrial strategy that would promote EU competitiveness and support the Commission's self-assigned "geopolitical" role by boosting strategic autonomy. In March 2020, while the COVID-19 pandemic was already dominating the public debate, the Commission adopted a Communication on "A New Industrial Strategy for Europe". In that document, the Commission observed that the "twin transition" (green and digital) was a unique opportunity for the EU to "affirm its voice, uphold its values and fight for a level playing field", adding that this "is about Europe's sovereignty" (European Commission, 2020, 1).

The Communication also stated that "Europe's industrial strategy must reflect our values and social market traditions" (European Commission, 2020, 1), which translates into an industrial policy focused on competition and open markets, rather than a revamp of protectionism or heavy subsidies to industry. Europe's values and traditions, of course, do not end with its unique approach to competition: they also extend to innovation, contracts, corporate governance and finance, as well as the emphasis on empowering small and medium-sized enterprises (SMEs), protecting fundamental rights and mainstreaming sustainability. The 2020 Communication did not venture systematically into these areas, but constantly referred to them.

The Commission also rightly argued that industry must play a leading role in helping the EU achieve climate neutrality by 2050: all value chains are involved, including existing ones and others to be launched through proactive policy aimed at boosting lead markets. Key pillars of the Communication included actions on strengthening the EU's specialisation in critical digital technologies such as 5G, artificial intelligence and metadata analytics; deepening the Single Market by adopting an SME-centric approach; revamping competition rules, including those on state aids; transitioning towards a circular economy; boosting innovation, *inter alia*, by consolidating and relaunching the European Innovation Council, and leveraging public-private partnerships to help industry develop the technologies to meet their goals; upgrading skills available to European industry; and financing investment and innovation.

The Communication also touched upon the external dimension of the EU industrial strategy by correctly observing that ambitious goals – in terms of sustainability, climate neutrality and even social policy – cannot be obtained if Europe fails to act to establish a level playing field with other countries. Key initiatives include the development of a deeper EU industrial base in strategic areas such as critical digital technologies, defence and space, and pharmaceuticals; and adopting legal and regulatory measures to rebalance global competition, including a screening mechanism for foreign investment, the enactment of a carbon border adjustment tax, and reinforcing customs controls.

The Communication also included a number of provisions related to governance, which, however, fell short of providing sufficient clarity on how the implementation of the industrial strategy would be ensured. The Commission announced several steps, including a focus on industrial ecosystems (14 clusters of sectors and value chains that the Commission has started to adopt as a way to "read" the European economy); the launch of a multi-stakeholder Industrial Forum, with the task of assisting the Commission in tracking the implementation of the industrial strategy and developing the work on ecosystems; and support for the instrument of industrial alliances, which already reportedly proved beneficial in areas such as batteries, plastics, microelectronics and hydrogen.

Against this background, however, the Communication fell short of laying the foundations for a complete set of governance arrangements, which would ensure at once coherence between the several actions foreseen in the strategy, and the achievement of clear, measurable and consistent impacts. The most evident gap was the lack of a set of indicators for monitoring and evaluating progress, an issue which was taken up swiftly by industry groups and associations, in an attempt to fill this gap. But beyond this, other governance challenges were left unaddressed: these include ensuring that EU lawmaking is designed to pursue the goals set by the Green Deal and the Industrial Strategy through a reorientation of the better regulation agenda; enabling a reform of corporate governance to boost systemic transformation; explaining how progress would be conceptualised and measured when adopting the "ecosystem" as the unit of analysis; and reconciling the ecosystems with the numerous other aggregations and clusters of industry actors used in the same or other areas of EU policy (e.g. data spaces, strategic value chains, alliances, Important Projects of Common European Interest, partnerships, missions, Knowledge and Innovation Communities, research infrastructures).

Some of these problems would have likely been addressed by the Commission in the months that followed the adoption of the Communication. However, a more serious set of events came to affect the agenda of the European Commission: the COVID-19 pandemic has indeed triggered a dramatic economic downturn and promises to leave an indelible mark on the future of the EU agenda, including of course the EU's industrial strategy.

### The pandemic changed everything, prompting a refocusing of the EU strategy

The European Union has already learned several hard lessons from the enduring coronavirus pandemic. It emerged quite clearly that the resilience of the Union is lowest where the competences of the EU are weakest, as in healthcare. The need to reorient the EU's action beyond areas originally identified as key priorities of the von der Leyen Commission also led to an overall shift in the focus of EU action during the first year of the pandemic.

In particular, the need to ensure greater resilience has now become the top priority for EU institutions, which has several consequences for EU industrial policy. While EU policy was aimed at "competitive sustainability" before the pandemic, the post-pandemic recovery is aiming at achieving both resilience and sustainability, which require bolder action on all fronts, including the systemic transformation of industry value chains; an ad hoc approach to digitalisation; a careful and pervasive mapping of the EU's dependencies on other powers, especially in key technologies and raw materials; and a renewed focus on mitigating the impact of the pandemic on labour markets, accelerating the up- and re-skilling of workers of sectors that are likely to experience the worst downturn.

Greater coordination, however, does not necessarily imply greater centralisation. The pandemic has also marked the rise of decentralised governance as both resilient and sustainable in many industrial settings. In particular, the decentralisation of decision-making and value distribution in complex value chains, as well as the adoption of more decentralised governance in the digital ecosystem (e.g. through edge computing) represent key new frontiers for the EU in the attempt to reconcile competition, coordination, efficiency, resilience and sustainability. Decentralised governance approaches also become an essential way to empower all those industry sectors which, with the (accelerated) digital transformation, are at risk of losing control of the value they generate. This is a concrete risk in several "ecosystems", from agriculture and food to energy, manufacturing, automotive industry and healthcare.

Despite a recent rebound of optimism among EU leaders, the projections for the EU are worse than for both the United States and China, which seem destined for an earlier recovery from the crisis as well as sustained growth in the medium term. Without disregarding concerns about the tentative nature of these estimates (as well as the questionability of GDP as a measure of prosperity), these prospects illustrate the uphill battle the EU will face in defending its geopolitical role in the global order. Some emphasise the danger of "scarring", i.e. a long-term impact of the pandemic on the economy of many countries. On the other hand, the crisis is also likely to accelerate change which might improve productivity growth (Kotz et al., 2021). The key to avoiding scarring while still reaping the benefits of change seems to be to ensure the stability of the financial system. This seems to be due in large part to the determined action of the ECB and the financial supervisory bodies.

When it comes to specific industries, a peculiarity of the COVID-19 pandemic is its extremely diverse impact across and within sectors, as shown by a recent study (de Vet et al., 2021). Industries like chemicals, construction, and the food and drinks sector are likely to experience a so-called V-shaped recovery from the crisis; whereas automotive and textile industries will likely be on an earlier recovery path. The worst impacts may occur in sectors that are dependent on human contact and interaction, such as the cultural and creative industries. Faced with such gloomy prospects, the EU has a moral and political imperative to approach the recovery by triggering a deep economic transformation, and to shift the whole direction of its action towards resilience and sustainability. The cornerstone of the EU post-pandemic strategy will inevitably be found in the Resilience and Recovery Fund: the stakes could not be higher, and the multi-level, public-private effort needed to restore a path to prosperity in Europe cannot be underestimated.

### The May 2021 Communication on updating the EU Industrial Strategy

In the new Communication, the European Commission (2021) reaffirms the priorities set out in the March 2020 Communication and devotes significant attention to the measures adopted to increase the resilience of the Single Market, including accelerating the work on the forthcoming regulation on foreign subsidies; adopting a Single Market Emergency Instrument to ensure the free movement of persons, goods and services in case of future crises; taking action to improve the implementation of the Service Directive; strengthening the market surveillance of products by supporting competent national authorities; mobilising significant investment to support SMEs with a dedicated SME Envoy, support from "sustainability advisors", alternative dispute resolution schemes and measures to address solvency risks.

Beyond the protection and enhancement of the Single Market, the Communication reports the results of a thorough mapping and analysis of Europe's strategic dependencies and "reverse dependencies", identifying 137 products in "sensitive ecosystems", for which the EU is highly dependent, showing weaknesses in energy-intensive industries, health and advanced technologies. However, these 137 products represent only a small share (6%) of total imports. The Commission presents six in-depth reviews on strategic areas, i.e. raw materials, batteries, active pharmaceutical ingredients, hydrogen, semiconductors and cloud and edge technologies. The resilience objective will be pursued also by taking action to diversify international supply chains and pursue international partnerships and alliances, in particular on processors and semiconductor technologies, industrial data, edge and cloud, space launchers, and zero emission aviation.

The work on the fourteen ecosystems showed in Figure 1 is coupled with initiatives aimed at co-creating "transition pathways" with industry, public authorities, social partners and other stakeholders, where needed, starting with tourism and energy-intensive industries. This is perhaps the most ground-breaking commitment included in the Communication. However, as recalled below, the extent to which this will configure a real modus operandi in the Commission is unclear at the time of writing.

All in all, the Communication shows a remarkable commitment to protecting the Single Market and promoting competitiveness, productivity and resilience in European industry. At the same time, its ambition does not go as far as realising the systemic change that the President of the Commission Ursula von der Leyen evoked with respect to the Green Deal. On the side of governance, the Industrial Forum launched in February 2021 seems to have fallen short of acquiring the centrality in governing the transition that it seemed to have gained in the run-up to the adoption of the Communication.

## Can the EU lead the way towards a new approach to industrial policy?

The European Commission should remedy the lack of ambition of the recent Communication on the update of the industrial strategy by taking six bold steps.

First, rather than a mere update, the Commission should adopt a full-fledged strategy, with a "North Star", i.e. a mission to be accomplished in the short and medium term and a concrete timeline, not only for the launch of specific initiatives, but also for the achievement of specific goals. Furthermore, the Commission should define concrete indicators, a governance framework and a contingency plan in case (some of the) progress towards some of the goals appears to be insufficient over time. Regarding indicators, the traditional focus on supply-side or input indicators (e.g. R&D investment as a percentage of GDP) should be complemented by a renewed attention

Figure 1

**Industrial ecosystems in Europe**

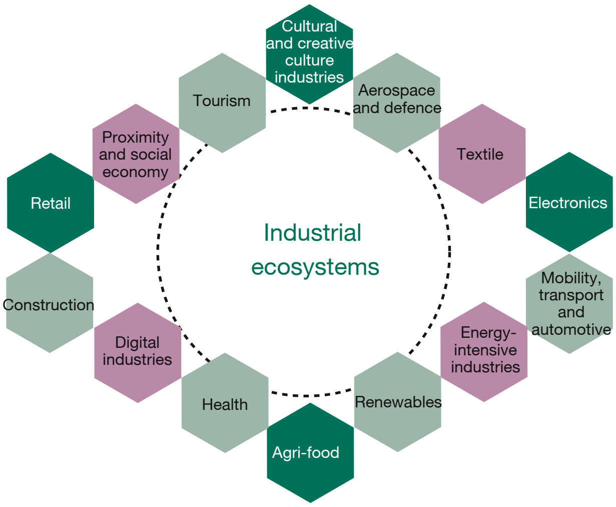

Source: European Commission (2021).

to output, outcome and impact indicators, linked to the overall North Star chosen for the strategy. And on governance, clear rules of engagement with the private sector and a contingency plan appear to be essential elements of a well-drawn strategy.

Second, in choosing its North Star, the Commission should embrace (a refined version of) the Industry 5.0 paradigm currently being developed by its Directorate-General for Research and Innovation. This also means recognising that the Industry 4.0 paradigm does not, in and of itself, provide a complete blueprint for industrial transformation, due to its limited attention for sustainability, let alone resilience and workers' well-being. The Industry 5.0 approach has the ability to potentially align industrial policy efforts with the overall agenda of the EU, surpassing the traditional separation between the state and the market, and charging both governments and the private sector with a shared responsibility to "row in the same direction", which would make strides in three main areas: building a human-centric industry focused on well-being, in particular, that of workers; fostering sustainability from an economic, social and environmental perspective; and enhancing resilience.

Third, the EU should firmly depart from unsustainable forms of capitalism that entail shareholder primacy. Even in the US, after resounding statements by the American Business Roundtable and by BlackRock's CEO among others, President Joe Biden openly committed to "put an end to the era of shareholder capitalism" (Plender, 2021). A new European Enterprise model should be explicitly based on the principles of fairness, resilience and sustainability (Kalff, 2021). Only in this way, industry can become the real engine of the "twin transition". More specifically: the sustainability pillar of Industry 5.0 requires enhanced corporate orientation towards the circular economy, mitigating climate impacts and ensuring fair value distribution along the value chains; and the resilience pillar requires more decentralisation in corporate organisation as well as a fairer value distribution in value chains.

Fourth, there is a need to consolidate and streamline the many initiatives launched to support industry at the EU level. At a minimum, the Commission should try to reconcile the notions of sector, ecosystem and data space to allow for a more coordinated approach to reaching medium- to long-term targets. After all, it would be very difficult to imagine a successful digital transformation in key ecosystems without a data governance strategy that matches the need, the actors and the value chains operating in those same ecosystems. Or, to put it differently, implementing a sectoral policy to achieve ecosystemic transformation would be preposterous.

Fifth, it is important to take Next Generation EU as a "once in a century" opportunity to rebuild, reshape and repurpose Europe's industry. The unprecedented resources made available to member states to "build back better", in the parlance of President Biden, should be used in a consistent and efficient way to avoid the aforementioned problems. System change cannot be achieved if Europe marches to the beat of two different drummers, one at the EU and another at the national level. The scrutiny, evaluation and implementation of national plans for recovery and resilience should therefore be given maximum attention and use new instruments rather than legacy, austerity-based tools. Merging abilities such as strategic foresight, knowledge on regional specialisation and the definition of pan-European industrial transformation pathways with key consequences for member states' agendas will be key to achieving the ambitions of the twin transition, as well as the "systemic change" recently evoked by Ursula von der Leyen (Sistemiq, 2020). Unfortunately, a first analysis of the available National Plans reveals that member states have given priority to measures aimed at "protecting" the economy and society, thereby mitigating the short-term effects of the pandemic rather than creating the preconditions for change. De Vet et al. (2021) observe that "most measures consisted of horizontal support instruments without predetermined focus"; they also mention the possible confusion generated by the multiplicity of targets given to national governments, including the twin transition, resilience, consistency with the country-specific recommendations, etc.; and the lack of meta-level coordination, especially the need to reconcile national plans with the need to proactively shape inclusive, resilient, fair and sustainable industrial value chains.

Finally, the choice of suitable indicators is of utmost importance. Continuing to track and reward corporate performance by referring to financial indicators and cost optimisation is likely to frustrate any attempt to embark on a system transformation towards Industry 5.0. Beyond the taxonomy (an essential piece of the puzzle), the EU industrial strategy and the various governance mechanisms it relies upon (Important Projects of Common European Interest, partnerships, public-private partnerships, missions, etc.) should measure progress way beyond mere inputs (e.g. R&D expenditure) and outputs (e.g. patent applications). Progress, however, is about outcomes and impacts, and EU institutions should be adequately equipped to measure those impacts on the ground, and take action when data show insufficient progress. As already mentioned, both the March 2020 Communication on "A New Industrial Strategy for Europe" and the recent update in the Communication on "Updating the 2020 New Industrial Strategy: Building a stronger Single Market for Europe's recovery" do not contain any concrete steps towards going beyond competitiveness indicators when tracking the progress of the industrial strategy.[1] Against this background, the proposed indicators developed by the European Roundtable of Industrialists (2020) already took a step forward in the addition of not only impact indicators but also more traditional input and output ones. The Commission, however, will need to develop articulate and comprehensive indicators mirroring the economic, social, environmental and governance pillars of the transition towards Industry 5.0, centred on well-being (and thus, inter alia on alternative measures to GDP); on resilience (as a further elaboration on the first dashboard developed by the Joint Research Centre); and on sustainability.
